# The differences in bioaccumulation and effects between Se(IV) and Se(VI) in the topmouth gudgeon *Pseudorasbora parva*

**DOI:** 10.1038/s41598-018-32270-z

**Published:** 2018-09-14

**Authors:** Shanshan Ma, Xiangfeng Zeng, Hongxing Chen, Shicong Geng, Liang Yan, Yongju Luo, Lingtian Xie, Qianru Zhang

**Affiliations:** 1 Key Laboratory of Pollution Ecology and Environmental Engineering, Institute of Applied Ecology, Chinese Academy of Sciences, Shenyang, 110016 China; 2 The Key Laboratory of Clean Combustion for Electricity Generation and Heat-Supply Technology, College of Energy and Power, Shenyang Institute of Engineering, Shenyang, 110136 China; 3 Department of Environmental Science, Zhejiang University, Hangzhou, 310058 China; 4 The Environmental Research Institute, MOE Key Laboratory of Theoretical Chemistry of Environment, South China Normal University, Guangzhou, 510006 China; 5 Institute of Applied Ecology, Chinese Academy of Sciences, Shenyang, 110016 China; 6 Guangxi Academy of Fishery Sciences, Nanning, 530021 China

## Abstract

Selenium (Se) might be protective against oxidative stress at nutritional levels, but elevated Se concentrations in the diet has been revealed as the main culprit for the extinction of natural fish populations in Se-contaminated lakes. Though Se predominate as waterborne selenite (IV) and selenate (VI) in the water, the differences in bioaccumulation, effects (e.g., oxidative stress, antioxidants etc.) and molecular mechanisms between Se(IV) and Se(VI) have been relatively understudied in wild fish. In this study, the *P. parva* were exposed to waterborne Se (10, 200 and 1000 μg/L of Se(IV) or Se(VI)) and sampled at 4, 14 and 28 days. Bioaccumulation, tissue distributions of Se and following effects in different tissues were evaluated. The results showed that the levels of Se in the gills and intestine were significantly elevated with a seemingly concentration-dependent pattern in the Se(IV) treatment, with respectively 173.3% and 57.2% increase after 28 days of exposure, relative to that of Se(VI) treatment. Additionally, significant accumulation of Se was also observed in the muscle of Se(IV) treated fish. Se exposure increased the MDA levels in the brain and gills in the Se(IV) treatment, but less apparent in the Se(VI) treatment. Meanwhile, Se exposure lowered (at least 56%) the activity of GST in the gills, but increased the activity of AChE in the muscle (~69%) and brain (~50%) after 28 d. Most importantly, after 28 d of exposure, Se exposure caused significant decrease in GSH levels in the gills (at least 35%) and in all tissues examined at the highest test concentration. In general, the results showed that Se(IV) led to faster accumulation of Se than Se(VI) in *P. parva*, and the resulted lipid peroxidation was closely related to the levels of antioxidants, especially GSH. Our results suggest that the ecotoxicological effects of waterborne selenite and selenate differ in this freshwater species in the field.

## Introduction

Selenium (Se) is an essential trace element for vertebrates including fish. In fish, Se is an important component for more than 40 selenoproteins which are essential to maintain normal physiology. However, it has a narrow margin between essentiality and toxicity, and becomes toxic to fish at a slightly elevated level beyond the optimum intake^1^. Selenium enters the aquatic ecosystems through multiple natural and anthropogenic processes^2^. Natural processes mainly include volcanic activity and weathering of rocks, while anthropogenic activities mainly include mining, fossil fuel combustion, oil refining, and irrigation of selenium-rich soils^3,4^. The background Se levels in water generally range from 0.1 to 10 μg/L^5^. Once in the water, selenite (i.e., SeO_3_^2−^ or Se(IV)), and selenate (i.e., SeO_4_^2−^ or Se(VI)) are the predominant forms of Se. Inorganic Se species could be biotransformed to organic Se (methylation) by primary producers existing in the aquatic environment.

Aquatic organisms including fish can take up Se via waterborne and dietary routes^6,7^. Once accumulated in organisms, the fate of inorganic Se and its essentiality and toxicity are determined by its complicated metabolism pathways^8^. Inorganic Se is first conjugated with GSH and then reduced to form H_2_Se. From there, Se can either be incorporated into selenoproteins (indicative of its essentiality for animals), reoxidized to SeO_2_ (during which reactive oxygen species is produced, suggestive of its toxicity to animals), or excreted in the form of selenosugar or methylated Se ((CH_3_)_3_Se^+^) in the urine^3^. Meanwhile, Se in the aqueous media and in the sediment can be transferred from low trophic level species (algae and benthos) to predatory fish and birds in the aquatic ecosystems^3^. The trophically transferred Se can provide essential Se nutrients required by the high trophic level species but can be toxic if this provision is more than required by these organisms^9–11^. It has been demonstrated that Se can be highly toxic to fish because it can rapidly accumulate and reach toxic level^1^. Field studies have shown that the extinction of natural populations of fish in Se contaminated lakes can be attributed to the elevated levels of Se in their diet. Fish exposed to high Se concentrations can be stressed by excessive ROS e.g., superoxide. It has been suggested that selenium-mediated thiol oxidation cause reactive oxygen species (ROS) and oxidative stress can be a factor related to selenium-induced toxicity^12,13^.

Aerobic organisms can prevent or limit cellular damage caused by ROS, and cells have evolved an interdependent antioxidant defense system. Among them, superoxide dismutase (SOD) decomposes superoxide anion to hydrogen peroxide, catalase (CAT) decomposes H_2_O_2_ to molecular oxygen and water, and glutathione peroxidases (GPx) reduce both H_2_O_2_ and lipid hydroperoxide. Glutathione (GSH) is the most plentiful intracellular thiol-based antioxidant, and function as a sulfhydryl buffer. It also has the function of detoxifying compounds via conjugation reactions catalyzed by glutathione S-transferases (GST). GST functions the detoxification enzyme existing all aerobic organisms, and catalyze the nucleophilic attack of the sulfur atom of the tripeptide glutathione^12,13^.

In addition, it was reported that although Se has neuroprotective effects^14,15^, it can also lead to neurotoxicity at high concentration Panter *et al*.^16^. Similarly, AChE activity could be inhibited in fish exposed to various toxic substances such as organophosphorus compounds, metals, and chemicals^17^. Indeed, the inhibition of AChE can be a biomarker for the neurotoxicity^18^.

Consequently, the study on the effects of dietary Se in aquatic species has been gained comprehensive attention^4,19^. Nevertheless, due to Se(IV) and Se(VI) are the main forms of Se and possibly the predominant Se species confronted in waterborne exposures by organisms in the aquatic ecosystems, the study on the bioaccumulation of aqueous inorganic Se in fish is relatively scarce.

The topmouth gudgeon *Pseudorasbora parva* is a small-sized freshwater cyprinid, originating from Northeastern regions of China. It has many attractive biological traits ideal for the ecotoxicological studies, including early maturity (sexually mature at 1 year), batch spawning, nest guarding and broad environmental tolerance limits^20^. Little is known about the bioaccumulation and effects of Se in this species. Previously, we showed that Se can rapidly accumulate in the liver of this fish via waterborne exposure^21^. The study’s main objective was to investigate the bioaccumulation, tissue distribution of Se and its effects on the antioxidant physiology. To achieve this, Fish (topmouth gudgeon *P. parva*) were exposed for 4, 14, and 28 d with waterborne Se concentration (0, 10, 200, and 1000 µg/L). The physiological effects of Se on the brain, gills, intestine and muscle were determined.

## Results

*P. parva* exhibited a mortality of 10% at 1000 μg/L Se(IV) treatment. In contrast, no fish mortality was observed in other Se treatments.

### Se accumulation in different tissue of *P. parva*

**Figure 1:**
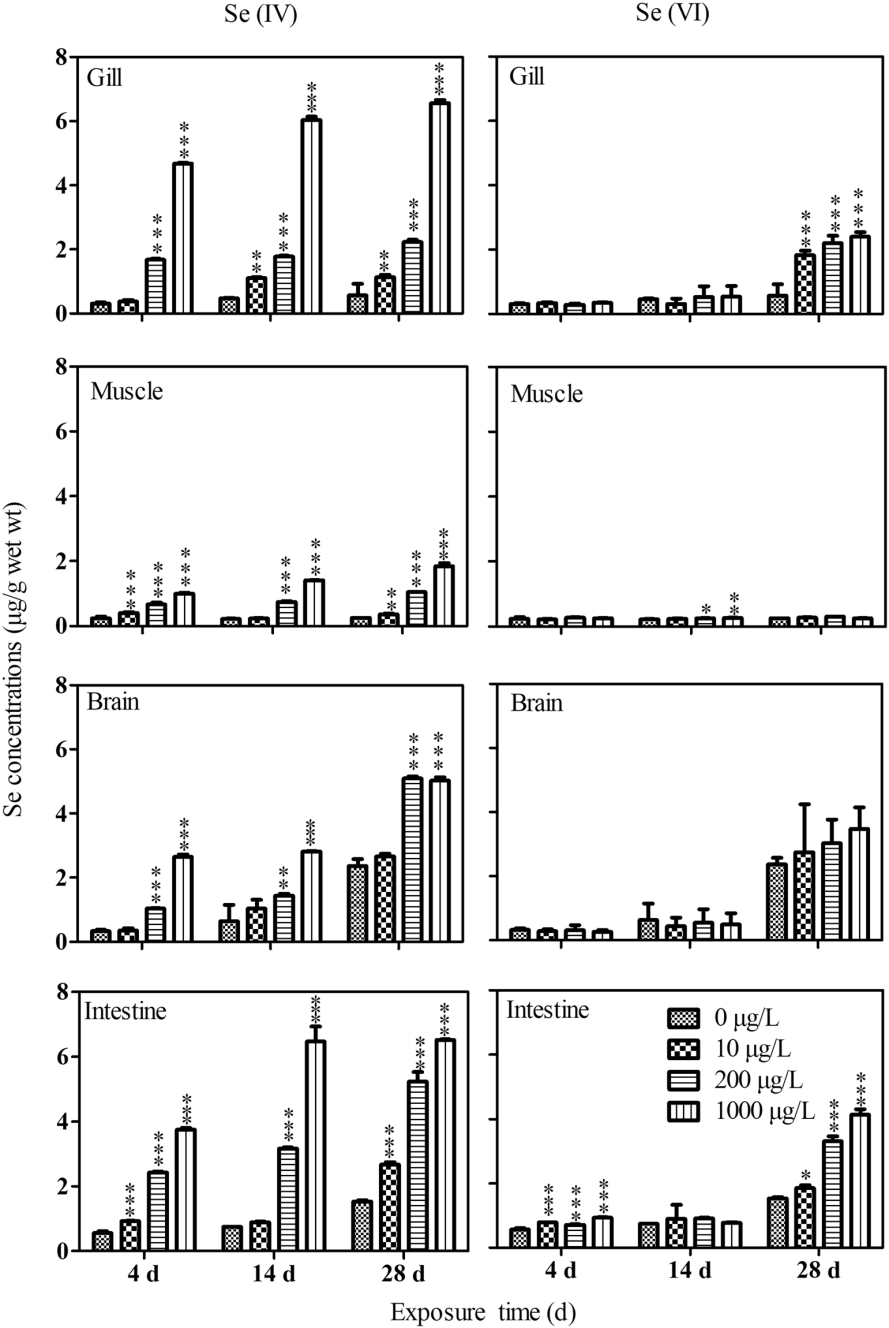
Se levels in different tissues of *P. parva* (*n* = 3) after exposure to dissolved Se for 4 d, 14 d, 28 d. Asterisks refer to statistical significance of comparison between the Se-exposed group and the control. *0.01 < *p* < 0.05, **0.001 < *p* < 0.01, ***p < 0.001.

Different patterns of Se accumulation among all tissues of *P. parva* were observed between these two Se species exposed groups (Fig. 1). For Se(IV), its accumulation in the tissues tested was concentration-dependent. In contrast, there was no obvious concentration-dependent Se accumulation in tested tissues after exposed to Se(VI) for 28 days. In short, tissues burdens of total Se in *P. parva* exposed to Se(IV) were higher than those of *P. parva* exposed to Se(VI) (*p* < 0.05). For example, after 28 d of Se exposure, gills burden of total Se in *P. parva* exposed to 1000 μg/L of Se(IV) were elevated by 173.3% than those of *P. parva* exposed to the same Se(VI) concentration (*p* < 0.05) (Fig. 1).

For Se(IV), Se levels in the gills, muscle, brain and intestine were significantly elevated during the exposure except for those in the gills and brain of *P. parva* at 10 µg/L Se(IV) treatment. Se levels in *P. parva* at 1000 μg/L after 28 d of exposure were 6.56 ± 0.09, 1.85 ± 0.09, 5.04 ± 0.10 and 6.51 ± 0.02 µg/g for the gill, muscle, brain and intestine tissues, respectively. The bioconcentration factor (tissue Se levels/aqueous Se levels) of Se was 6.1, 1.7, 4.7 and 6.0 for the gill, muscle, brain and intestine, respectively. In addition, higher Se(IV) levels (i.e., 200 and 1000 µg/L) of exposure led to similar rates of Se bioaccumulation in gills, muscle, and intestine.

For Se(VI), bioaccumulation in different tissues was not very obvious though significant differences were observed occasionally in the intestine and the muscle after exposure to Se for 4 and 14 days (Fig. 1). Se accumulation from exposure to Se(VI) was significantly elevated in the gills and the intestine but not in the brain and the muscle after 28 d of exposure. Gills burden of total Se in *P. parva* exposed to 10, 200, and 1000 μg/L of Se were about 226.8%, 292.9%, 328.6% higher (*p* < 0.05) than those of the control gills, respectively. Intestine burden of total Se in *P. parva* exposed to 10, 200, and 1000 μg/L of Se were elevated by 21.2%, 116.3%, 170.6% (*p* < 0.05) than those of the control intestine, respectively. The bioconcentration factor for Se(VI) in the gills and intestine after 28 d of exposure was 2.4 and 4.1, respectively. No significant differences were found in brain burdens of total Se in *P. parva* exposed to Se(VI) on the same sampling day (Fig. 1).

### Effects of Se on MDA (lipid peroxidation) and antioxidants in the tissues

**Figure 2:**
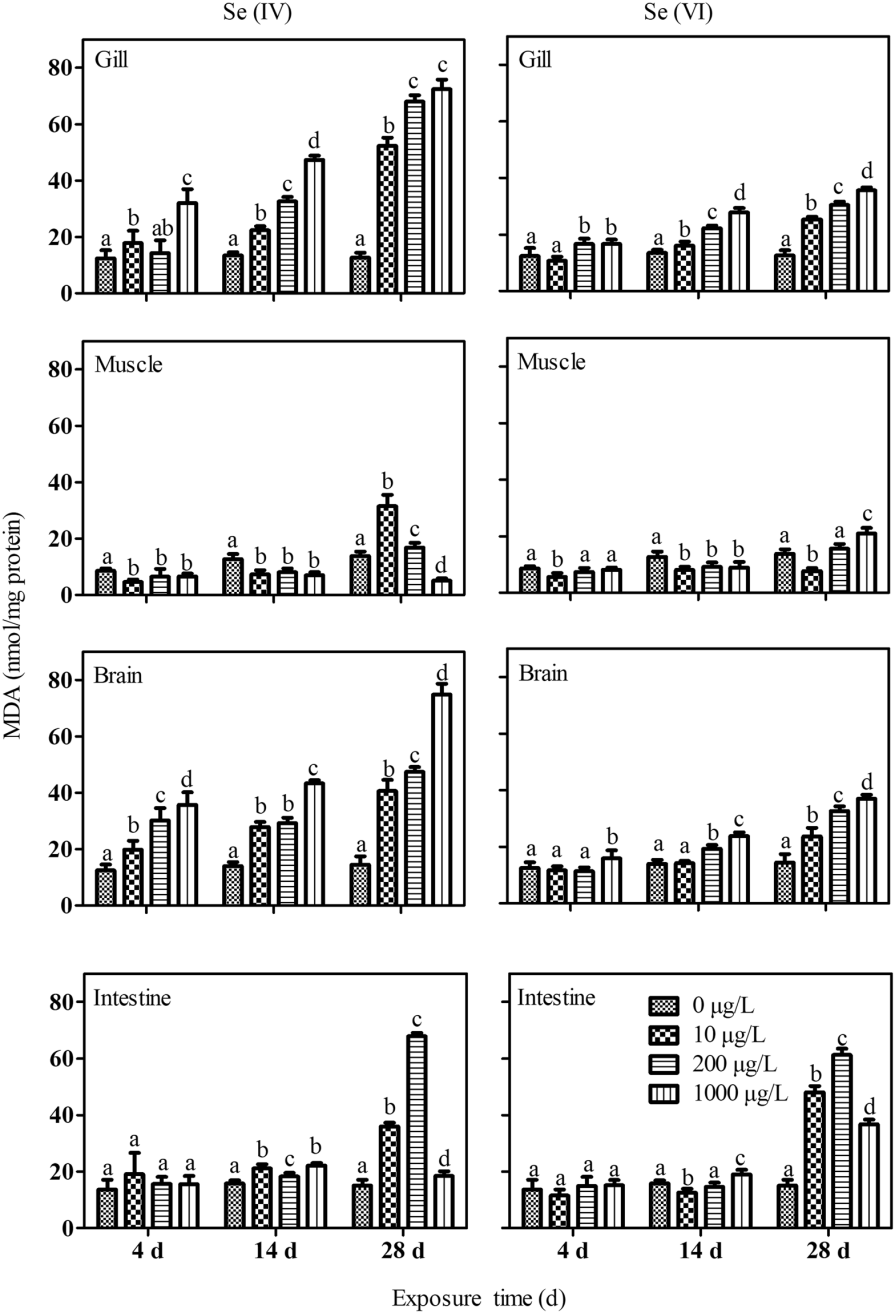
The effects of Se on malondialdehyde (MDA) level in different tissues of *P. parva* (n = 6). Within each concentration dependent variable, values with different letters are significantly different (*p* < 0.05).

Exposure to Se(IV) caused concentration-dependent increase in MDA levels in the gills after 14 and 28 d of exposure. A concentration dependent of increase of MDA levels to exposure to Se(IV) was observed in the brain on all sampling days (Fig. 2). On the contrary, in the muscle, MDA levels were significantly decreased after 4 and 14 days of exposure. However, after 28 days of exposure, MDA levels in the muscle and in the intestine were in general elevated with the exception of a decrease in the muscle of the fish at 1000 µg/L Se(IV) treatment. Exposure to Se(VI) caused a concentration-dependent increase in MDA levels in the fish gills (Fig. 2). Increased levels of MDA were observed in the fish brain after 14 and 28 days of exposure. Increased levels of MDA were observed in the intestine of fish after 28 d of exposure at all exposure concentrations (Fig. 2). In the muscle, decreased MDA levels were observed after 14 d of exposure, while elevated MDA levels were found only in *P. parva* at 1000 µg/L Se(VI) treatment (Fig. 2).

**Figure 3:**
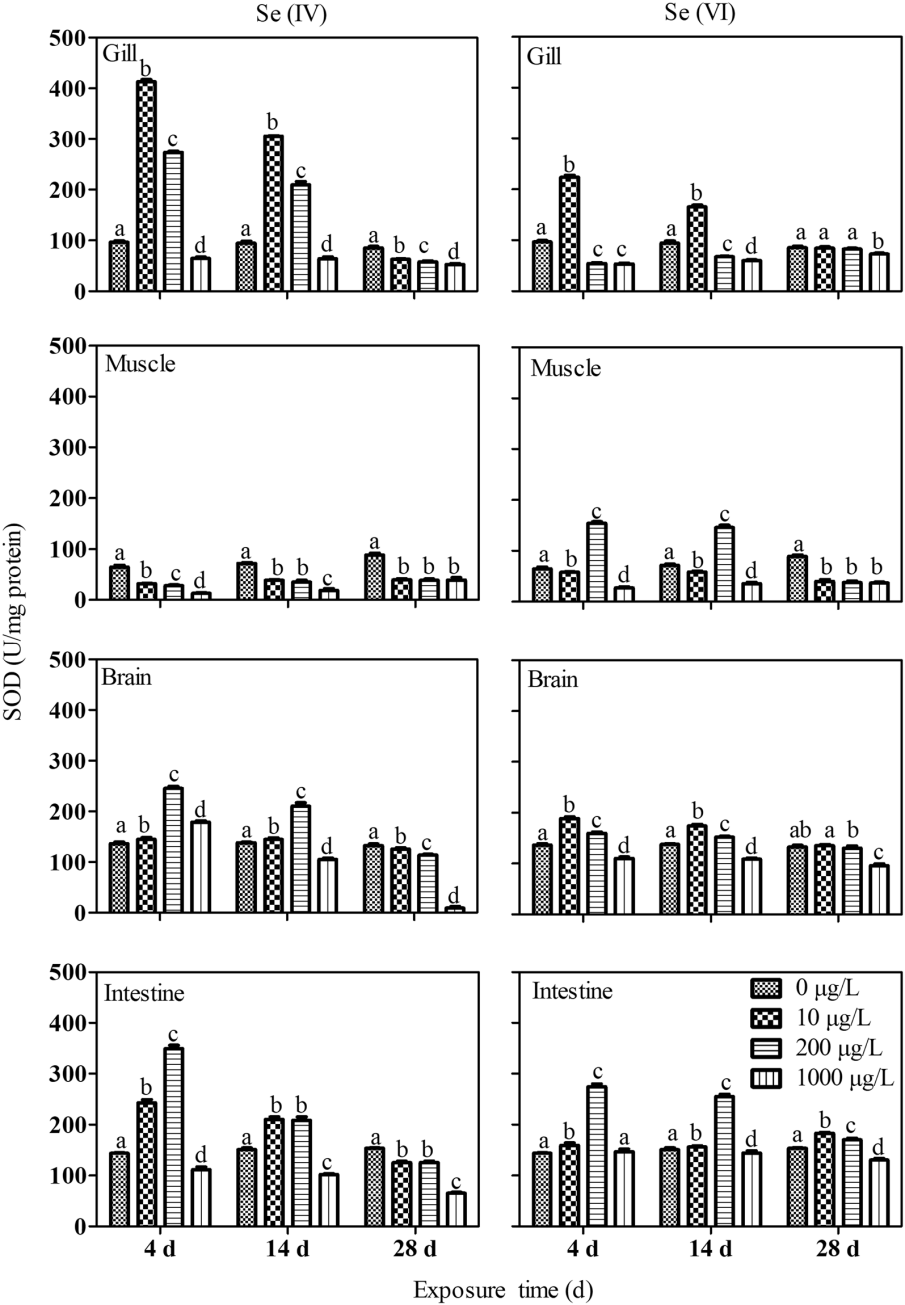
Response of superoxide dismutase (SOD) in different tissues of *P. parva* (n = 6) exposed to different concentrations of Se(IV) and Se(VI). Within each concentration dependent variable, values with different letters are significantly different (*p* < 0.05).

The activity of SOD was stimulated in the gills of fish at 10 and 200 µg/L of Se(IV) exposure for 4 and 14 d, but was inhibited at 1000 µg/L. Whereas the activity of SOD in the gills after 28 d of exposure was decreased in a seemingly concentration-dependent pattern (Fig. 3). SOD activity was in general inhibited in the muscle (Fig. 3). SOD activity in the brain showed a similar response to that in the gills, while SOD activity in the intestine exhibited a similar pattern to that in the muscle. SOD activity in the gills of Se(VI) exposed fish was increased at 10 µg/L Se(VI) after 4 and 14 d of exposure but decreased at 1000 µg/L Se(VI) after 28 d of exposure. In the muscle, inhibition of SOD activity was observed in fish from all treatments after exposed for 28 days. In general, the activity of SOD was decreased in the brain and in the intestine of the fish at 1000 µg/L Se treatment on all the sampling days.

**Figure 4:**
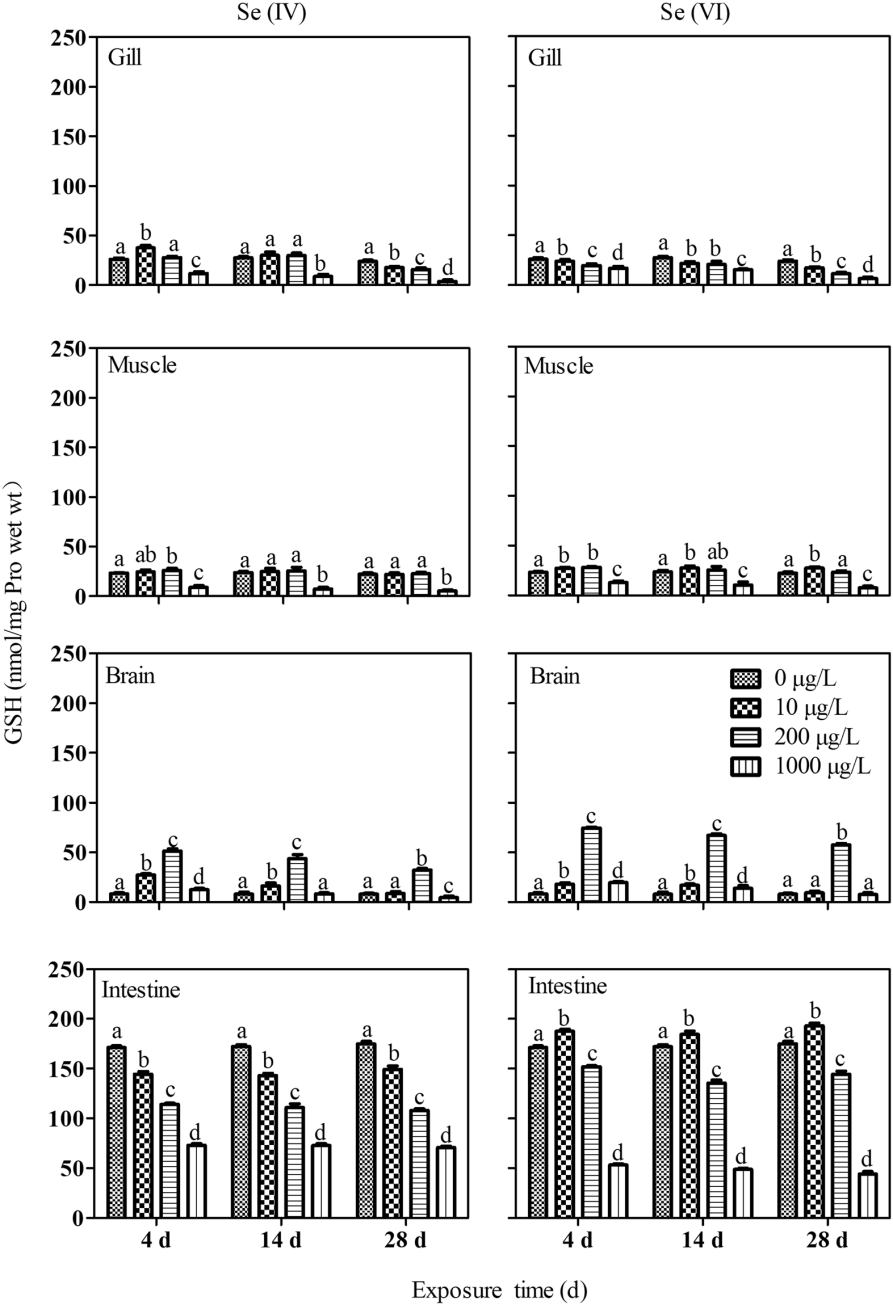
Response of glutathione (GSH) in different tissues of *P. parva* (n = 6) exposed to different concentrations of Se(IV) and Se(VI). Within each concentration dependent variable, values with different letters are significantly different (*p* < 0.05).

Compared with the control, after 4 and 14 d of exposure, the level of GSH in the gills of *P. parva* in Se(IV) treatment was significantly increased at 10 µg/L but decreased at 1000 µg/L. GSH level was significantly decreased in fish from all treatments after 28 d of exposure (Fig. 4). In general, GSH level was decreased at 1000 µg/L on all sampling days but increased at 200 µg/L Se treatment after 14 d of exposure. GSH in the brain was increased in fish from the 200 µg/L on all sampling days but was decreased at 1000 µg/L after 28 days of exposure. In the intestine, GSH levels showed a consistent concentration-dependent decrease on all sampling days (Fig. 4). GSH levels in the gills of Se(VI) exposed fish was generally decreased (Fig. 4). In the muscle, GSH level was decreased at 1000 µg/L Se(VI) treatment but increased 10 µg/L on all sampling days. The level of GSH in the brain of Se(VI) exposed fish was increased after 4 and 14 days of exposure and at 200 µg/L Se treatment after 28 d of exposure. GSH level in the Se(VI) treatment was increased in *P. parva* at 10 µg/L on all sampling days but was decreased at all other concentrations.

**Figure 5:**
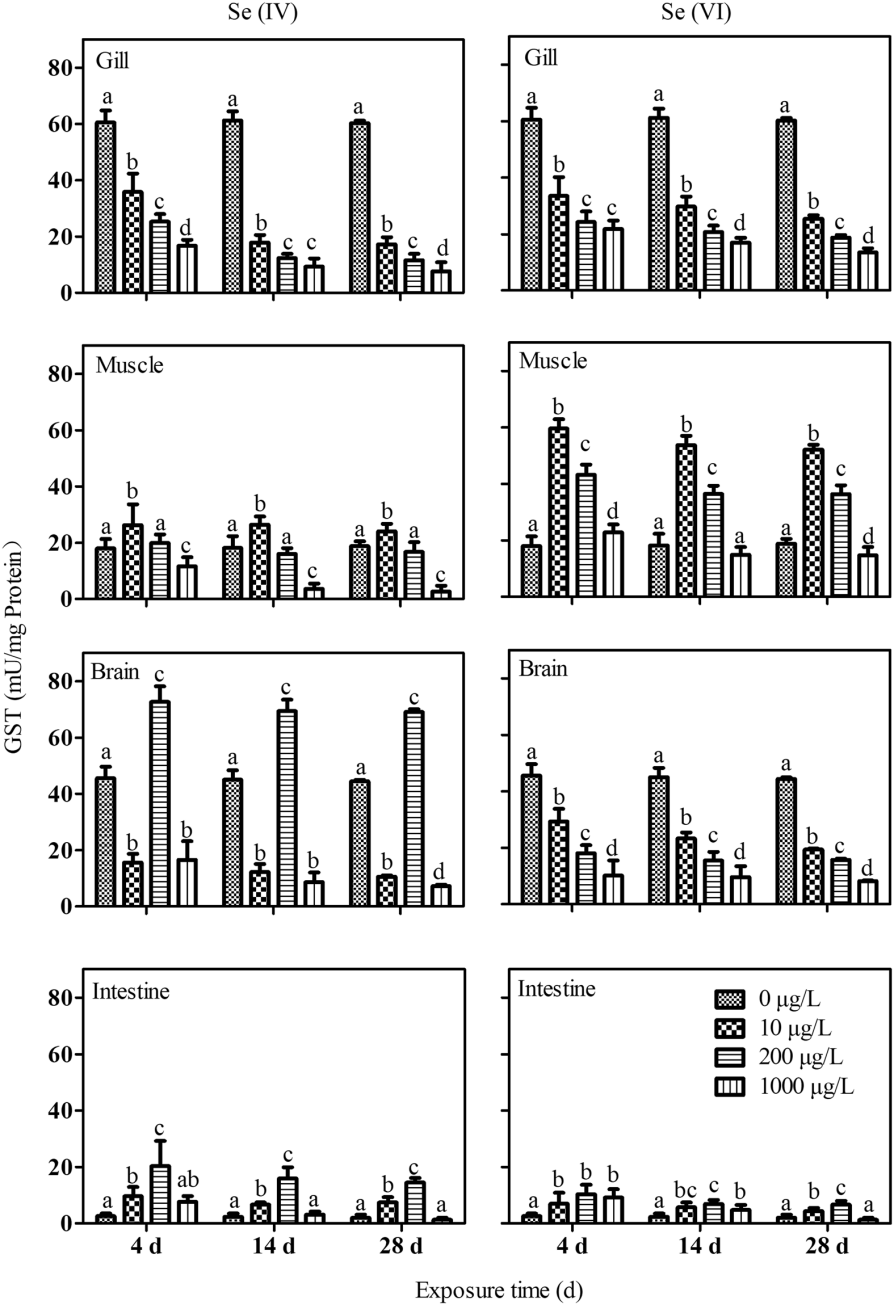
Response of glutathione-*S*-transferase (GST) in different tissues of *P. parva* (n = 6) exposed to different concentrations of Se(IV) and Se(VI). Within each concentration dependent variable, values with different letters are significantly different (*p* < 0.05).

In general, GST activity was significantly decreased in the gills of *P. parva* exposed to these two Se species at all concentrations and on all sampling days (Fig. 5). However, GST activity was often increased in the intestine and muscle of *P. parva* and was only decreased at highest test concentration (i.e., 1000 µg/L) of both Se exposures. In the brain, GST activity was increased at 200 µg/L Se(IV) treatment but decreased at other exposure concentrations of Se(VI) and Se(IV) (Fig. 5).

**Figure 6:**
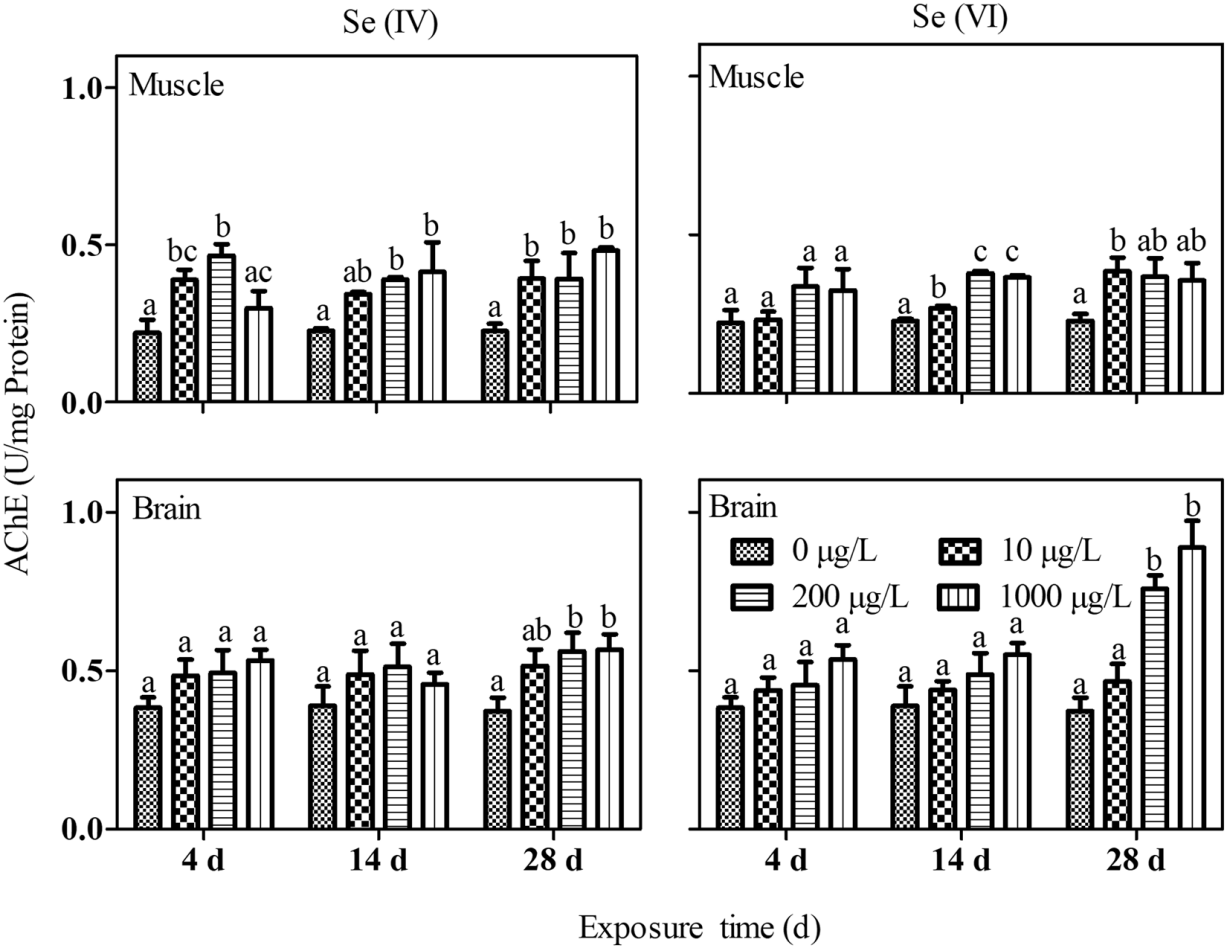
The effects of Se on acetylcholinesterase (AChE) activity in brain and muscle tissues of *P. parva* (n = 6) exposed to different concentrations of Se(IV) and Se(VI). Within each concentration dependent variable, values with different letters are significantly different (*p* < 0.05).

In general, AChE activity was increased after Se exposed for 28 d but remained relatively unaffected after 4 and 14 d of exposure (Fig. 6). In the muscle, AChE activity was in general increased by Se(IV) after 14 and 28 days of exposure. AChE activity was increased at 200 and 1000 µg/L Se(VI) exposures for 4 and 14 d, and at 10 µg/L Se(VI) exposure for 28 d (Fig. 6).

## Discussion

Bioaccumulation of total Se was significantly higher in the gills and intestine tissues of waterborne Se-exposed fish than those in the control fish after 28 d of exposure. Moreover, Se accumulation was apparent in the brain and in the muscle after Se(IV)-exposed for 28 d at the two exposure concentrations (200 and 1000 µg/L), while its accumulation was not obvious in these tissues after exposure to Se(VI) for 28 d. In addition, it seemed that 200 and 1000 µg/L Se(IV) exposure led to similar rates of Se bioaccumulation in gills, muscle, and intestine of *P. parva*. These results demonstrated that in general Se accumulation was more rapid from exposure to waterborne Se(IV) than that from waterborne Se(VI). Accordingly, the bioconcentration factor was also much higher for Se(IV) than for Se(VI) in *P. parva*. The general accumulation trend is faster for Se(IV) than for Se(VI), which is in agreement with that from previous studies. Our previous study showed that Se accumulation from waterborne Se(IV) exposure was higher than that of Se(VI) in the oligochaete *Lumbriculus variegatus* (15 µg/L for 2 weeks)^4^. Similarly, the accumulation of Se from waterborne Se(IV) (3.8 µg/L) exposure showed a time-dependent pattern, while accumulation of Se from Se(VI) was negligible during a 10 d exposure. The accumulation of Se from Se(IV) was approximately 7-folds of that from Se(VI) exposure after 10 d in midge *Chironomus dilutes* (Diptera: Chironomidae)^7^. The faster accumulation of Se from Se (IV) than Se(VI) also holds true in the dietary exposure experiments when aquatic organisms were exposed to dietary Se^4,22^. All these results suggest that selenite has higher bioavailability than selenate^23^. In short, Se(VI) can be reduced to Se(IV) and then to elemental selenium and Se(-II). The transformation rate of Se(IV) tends to be higher than that of Se(VI) during series of reduction^24^. This might account for the faster bioaccumulation of Se(IV) than Se(VI) in fish in this study.

Bioaccumulation of total Se was more apparent in the gills and in the intestine for these two Se species. Gills are the first organs in contact with Se during the aqueous exposure, therefore the organ most likely to have the largest accumulation. However, Se levels in the intestine were very comparable to those in the gills in this study. This could be due to the chemical property of Se element. It is suggested that Se could be redistributed to intestine via gills and blood circulation^25^. In addition, it could also be due to the anionic character of Se since cationic metals might have higher retention in the gills because of mucous precipitation in the gills^25^. Interestingly, an appreciable amount of Se was found in the muscle of Se(IV) exposed *P. parva*. It is generally believed that Se(IV) has a high affinity for free sulfhydryl groups on many organic molecules in fish skeletal muscle, including simple amino acids, peptides, and low molecular weight proteins^26^. In addition, Se is an essential metal which can be incorporated into proteins in a non-specific way^27,28^. Previous studies have demonstrated that Se is frequently found in fish muscle^29,30^. The occurrence of Se in the fish muscle might reflect its essentiality for animals.

In general, Se levels in different tissues were comparable to those from previous studies. For instance, after Se-exposed fish at 10 μg/L for 28 d, total Se levels in the gills and muscle of *P. parva* were 1.14 and 0.35 µg/g (wet wt) in waterborne Se(IV)-exposed topmouth gudgeon (*P. parva*) and 1.83 and 0.27 µg/g (wet wt) in waterborne Se(VI)-exposed topmouth gudgeon (*P. parva*), respectively. After Se(IV)-exposed fish at 10 μg/L for 30 d, total Se levels in the gills and white muscle of the bluegill (*Lepomis macrochirus*) were 6.6–9.4 and 0.7–1.2 µg/g (wet wt), respectively, and 6.0–8.4 and 0.3–0.9 µg/g (wet wt) in the gills and white muscle of largemouth bass (*Micropterus salmoides*), respectively^25^. After Se(IV)-exposed fish at 43.2 μg/L for 14 d and 28 d, total Se levels in the muscle of Tilapia fish (*Oreochromis spp*.) were 7.2–7.7 µg/g and 8.3–9.4 µg/g, respectively^6^. After Se(VI)-exposed fish at 4.8 μg/L, Se(IV)-exposed fish at 3.8 μg/L, and SeMet-exposed fish at 1.8 μg/L for 10 d, the Se concentration in midge *Chironomus dilutus* (Diptera: Chironomidae) was 2.1 µg/g, 14.5 µg/g, and 32.2 µg/g (dry wt), respectively^7^. After dietary Se(IV)-exposed fish at 15.01 μg/g for 12 weeks, the Se concentration in the intestine of rainbow trout (*Salmo gairdneri*) was 7.6–9.8 µg/g dry wt^31^. The difference in the level of Se accumulation may be due to species specificity. The source of Se (dietary vs waterborne) may also influence the bioaccumulation of Se via regulation of excretion rates by affecting the form of Se stored in tissues^31^. Most importantly, the levels of Se in the tissues (e.g., gills and intestine) of *P. parva* (5–7 µg/g) after Se-exposed fish at 1000 µg/L for 28 days in this study were accessing the values in the liver tissues of bluegill from a Se-contaminated lake (Hyco Lake)^3^. Therefore, it is likely that Se could also cause adverse effects in *P. parva*.

Exposure to excess Se can result in a notable increase of lipid peroxidation in fish^4,32^. Malondialdehyde (MDA) highly represented among the products of lipid peroxidation^33^. Lipid peroxidation is oxidative damage that attack all cellular constituents. It can increase the permeability of cellular membranes, leading to important changes in membrane function. It also precedes irreversible cell damage/death^34^. In present study, the levels of MDA were significantly raised in the brain, gill and intestine after Se-exposed *P. parva* at 1000 μg/L for 14 and 28 d. Increased lipid peroxidation has been frequently observed in fish exposed to Se^4,35^. It is commonly considered that the shaping of CH_3_Se^−^ during Se speciation in the cytoplasm is closely associated with the following production of lipid peroxidation and superoxide in organisms^36^. In addition, it is shown that intracellular Se(IV) is capable of generating superoxide anion radical (O_2_^−^)^37^. It is likely that elevated Se exposure caused excess ROS, surpassing the scavenging capacity of the enzymatic and non-enzymatic antioxidants. This can then lead to the observed lipid peroxidation^34^. Furthermore, the present study showed that the SOD activity was inhibited in the fish exposed to both Se species, especially after 28 d of exposure. This suggested that although Se is an important component of GPx, it might inhibit the activity of other antioxidant enzymes. Interestingly, MDA levels were significantly lower in the muscle of Se(IV)-exposed fish than control, suggesting the beneficial role of Se at nutritional level, which might be due to the limited accumulation of Se in this tissue. Similar results were found in the muscle of rainbow trout and least killifish exposed to low level of dietary Se^4,38^.

The SOD activity in tissues of *P. parva* were generally stimulated at lower Se levels but inhibited at elevated Se levels. Similar results have been reported in a previous study showing that the activity of SOD was notably enhanced in the liver tissues after Se(IV)-exposed rainbow trout at 900 and 1800 μg/L for 3 d, but maintained the control levels at the concentration of 2700 μg/L. In addition, prolonged exposure to Se also resulted in lower SOD activity in Japanese medaka (*Oryzias latipes*)^39,40^. However, lipid peroxidation was observed in various tisues of *P. parva* in the present study, indicating the change of SOD activity in this fish species might not be sufficient to detoxify the excess ROS generated by Se exposure. GSH levels in the tissues of *P. parva* were generally decreased after exposure to Se, especially at 1000 µg/L treatment and after 28 d of exposure. This is in consistence with a previous study showing that the GSH concentration in the liver tissues after Se(IV)-exposed juvenile rainbow trout (*Oncorhynchus mykiss*) at 2500–3600 μg/L for 4 d was significantly decreased^12^. GSH occupies a central role in preserving organisms from multiple contaminants by conjugating contaminants with thiol group (SH)^41,42^. The shortage of GSH contributed to the over-production of ROS^34,37,41,42^, which was also observed indirectly in this study. It was reported that GSH could participate in metabolic transformation of both Se species (i.e., Se(VI) and Se(IV)) once involved into the different tissues of fish^8^. Meanwhile, it is believed that Se(IV) could be reduced by GSH into H_2_Se inside cells^43^, accounting for the increased level of lipid peroxidation in fish exposed to both Se species in present study. Alteration of GST activity in the tissues of *P. parva* was not consistent except in the gills of the fish, where GST activity was generally significantly decreased. Previously it was reported that the activity of GST in liver tissues of rainbow trout could be inhibited by dietary Se(IV)^44^. GST is an indemnificatory element of a selenoprotein (i.e., glutathione peroxidase or GPx)) against superfluous ROS and its activity expression can be conducted by Se^45^. The inhibited GST levels in the gills implied the redox reaction between Se and GST, therefore resulting in surplus production of ROS^34,41,42^. The inconsistency of the change of GST activity in other tissues suggested that GST might play a minor role in the detoxification of ROS in these tissues. Nonetheless, early findings have demonstrated that the activity of GST was always inferior in organisms after dietary Se exposure, combined with a relatively higher level of GPx^44^. This might be due to the differences between dietary and waterborne exposure.

Se has neuroprotective effects at trace levels^14,15^, whereas itself can lead to neurotoxicity at high concentration^16^. AChE is a member of the enzyme family known as ChE and is responsible for degrading the neurotransmitter acetylcholine in cholinergic synapses^46–49^. In this study, in general, the AChE activity of *P. parva* was increased in the brain (more obvious) and muscle of *P. parva* after both waterborne Se(IV) and Se(VI) exposures. However, it was reported that the AChE activities in the brain and muscle tissues were decreased after Se(IV)-exposed red sea bream (*Pagrus major*) at 400 µg/L for 28 d^13^. This difference might reflect species specificity on the role of Se in AChE. In general, the effect of Se on AChE in fish has been relatively less studied and future research is warranted.

## Conclusions

The present study showed that the bioaccumulation and effects of Se in different tissues of topmouth gudgeon *P. parva* from waterborne exposures of both Se species (i.e., Se(IV) and Se(VI)) cannot be overlooked. The levels of antioxidant enzymes, GSH, and lipid peroxidation vary with total Se level closely in different tissues of *P. parva*. Our findings make it obvious that Se(IV) has higher bioavailability and more toxic to *P. parva* than Se(VI). In view of relatively short exposure duration in present study, the potential threats submitted by waterborne Se-exposed organisms in the wild may be much more serious because of its life-long exposure to aqueous Se.

## Methods

### Chemicals and reagents

Selenite (or Se(IV), in the form of Na_2_SeO_3_) and selenate (or Se(VI), in the form of Na_2_SeO_4_) were purchased from Sigma-Aldrich. All containers were acid-washed with 10% nitric acid, rinsed with deionized water and oven-dried at 40 °C before use.

### Ethics statement

All the methods used in this study were performed in accordance with the Guidelines for Experimental Animals established by the Ministry of Science and Technology (Beijing, China). The study protocols were approved by the Institutional Animal Care and Use Committee of Shenyang Institute of Applied Ecology (IAE), Chinese Academy of Sciences and the China Government Principles for the Utilization and Care of Vertebrate Animals Used in Testing, Research, and Training (State science and technology commission of the People’s Republic of China for No. 2, October 31, 1988: http://www.gov.cn/gongbao/content/2011/content_1860757.htm).

### Test animals

*P. parva* were obtained from a local market. Approximately 1000 topmouth gudgeon *P. parva* were acclimated to test conditions for at least 2 weeks prior to exposure. The fish (weight: 735.4 ± 32.5 mg, standard length: 4.70 ± 0.60 cm) were fed newly hatched *Artemia* nauplii once daily.

### Se exposure

The experiment included seven treatments and each concentration (control and treatment) had 3 replicates. For each replicate, 40 fish were placed in plastic aquaria with 40 L of reconstituted medium hard water. The measured concentrations of NaHCO_3_, CaSO_4_, MgSO_4_ 7H_2_O, and KCl were 96 mg/L, 47.5 mg/L, 123.0 mg/L, and 4.0 mg/L, respectively. The exposure media were changed totally once every two days and six water samples (each 20 mL) were immediately taken before (3) and after (3) the water renewal for the determination of Se concentrations in the water. The determination of Se concentration in the water was only processed in the first week.

The exposure lasted for 28 d. The measured water quality parameters were not significantly different (*p* > 0.05) among the waterborne exposure. During the trial, water temperature, light: dark, pH, dissolved organic carbon (DOC), dissolved oxygen (DO), and electrical conductivity were 25 ± 1 °C, 16 h: 8 h, 8.02 ± 0.16, 2.1 ± 0.3 mg/L, 8.98 ± 0.12 mg/L, and 271 ± 15.6 μs/cm, respectively (mean ± S.E., n = 3). Total ammonia (NH_3_/NH_4_^+^) was always ≤1.0 mg/L. The Se concentration in each tank was monitored before and after each water renewal, and concentrations in the control and treatment aquaria were 0 (control), 9.3 ± 0.5, 242.9 ± 20.3, 1076.3 ± 10.1 μg/L for Se (IV) and 9.3 ± 0.4, 184.9 ± 2.5, 1011.7 ± 35.2 μg/L for Se (VI), respectively (n = 3). The Se level in the *Artemia* nauplii was 0.78 ± 0.15 μg/g (wet wt, n = 3).

12 fish from each treatment were sampled after 4, 14, and 28 days of exposure. The fish were sacrificed on ice. Brain, gills, intestine, and muscle tissues were obtained, weighed, flash-frozen in liquid nitrogen, and stored at −80 °C for subsequent Se accumulation and biochemical analysis.

### Biochemical analysis

The activities of antioxidants were determined following methods in previous studies^21,50–54^. The superoxide dismutase (SOD) assay was performed according to Sun *et al*.^51^. The glutathione *S*-transferase (GST) was determined following Habig *et al*.^50^. The reduced glutathione (GSH) was measured according to Shaik and Mehvar^52^. The acetylcholinesterase (AChE) was determined according to Assis *et al*.^53^. Malonaldehyde (MDA) levels were determined by the content of thiobarbituric acid reactive substances (TBARS)^54^. For calculating enzyme activities, it was normalized to total protein concentration. Enzyme activities were determined as mU/mg total protein^53^ and the total protein was evaluated by the Bradford method^55^. Details for each assay are provided in the Supplementary File S1.

### Total Se determination

The gills, muscle, brain, and intestine tissues were digested in centrifuge tubes (15 mL capacity) with concentrated nitric acid (5 mL, 70%) in an oven at 80 °C for 2 h. After cooling, hydrochloric acid (2 mL) was added to oxidize Se(IV) to Se(VI) in the samples and heated at 90 °C for 1 h. The sample in each tube was diluted with DI water to a final volume of 10 mL. The samples were analyzed using an Atomic Fluorescence Spectrometer (AFS) (Haiguang, Beijing). Three replicates of the standard reference material (SRM) (i.e., GBW10024 scallop sample) from the National Research Center for Certified Reference Materials were used for Se. The recovery for Se in the SRM was approximately 95–110%. Other QA/QC samples included spiked samples and acid blanks. Blanks were analyzed at a rate of 1 per 5 samples.

### Statistical analysis

All of the final data were expressed as mean ± standard deviation (SD), unless otherwise stated. Data were checked for normality and homogeneity of variances using Kolmogorov-Smirnov test and Levene’s F test, respectively. No significant deviations from normality and homoscedasticity were detected. Analysis of variance (ANOVA) was used to assess the presence of differences among treatment groups. Tukey’s test was used for multiple comparisons among treatments. All statistical analyses were performed on Graphpad Prism software (version 5).

## Electronic supplementary material

Supplementary File S1

## Data Availability

Data are available for those who are interested on request (xielt@iae.ac.cn). Most data are in Excel files. Analyzed data are in Prism format.
