# Supplementary material for: The differences in bioaccumulation and effects between Se(IV) and Se(VI) in the topmouth gudgeon Pseudorasbora parva
Source: Sci Rep. 2018 Sep 14;8:13860. doi: 10.1038/s41598-018-32270-z (PMC6138650; doi:10.1038/s41598-018-32270-z)
Supplement: Supplementary file 1 — Supplementary File S1 [file 41598_2018_32270_MOESM1_ESM.docx]

**The differences in bioaccumulation and effects between Se(IV) and Se(VI) in the topmouth gudgeon *Pseudorasbora parva***

Shanshan Ma^1,2a^, Xiangfeng Zeng^1,3a^, Hongxing Chen^4^, Shicong Geng^5^, Liang Yan^1,2^, Yongju Luo^6^, Lingtian Xie^4^ and Qianru Zhang^1^*

1. Key Laboratory of Pollution Ecology and Environmental Engineering, Institute of Applied Ecology, Chinese Academy of Sciences, 110016, China

2. University of Chinese Academy of Sciences, Beijing 100049, China

3. Department of Environmental Science, Zhejiang University, Hangzhou, Zhejiang 310058, China

4. The Environmental Research Institute, MOE Key Laboratory of Theoretical Chemistry of Environmental, South China Normal University, Guangzhou 510006, China

5. Institute of Applied Ecology, Chinese Academy of Sciences, Shenyang 110016, China

6. Guangxi Academy of Fishery Sciences, Nanning, Guangxi 530021, China

^a^ These authors contributed equally to this work

* Corresponding author：Qianru Zhang, Email address: [zhangqianru@iae.ac.cn](mailto:zhangqianru@iae.ac.cn)

All enzyme assays were performed in a 96 well plate using a Thermo Multiskan FC microplate reader according to the methods from our earlier studies ^1-3^. For calculating enzyme activities, it was assumed that one unit of enzyme activity equals the amount of enzyme capable of consuming 1 µmol of the substrate per minute. Enzyme activities were determined as U mg total protein^-1^ ^1^ which was determined according to the Bradford method with bovine serum albumin as the standard.

For the determination of SOD and GST activities, the tissues were thawed on ice and homogenized in 50 mM phosphate buffer (pH 7.4, with 1 mM EDTA) using a Pestles in G-Tubes^®^ 1.5-mL homogenizer (Thomas Scientific). The homogenates were centrifuged (10,000 × g) at 4°C for 10 min. The SOD activity was measured using the method of Sun et al. ^4^. Briefly, 100 µL of SOD standards or sample solution was mixed with 100 µL of 50 µM nitro-blue tetrazolium chloride (NBT) and 40 µl of xanthine oxidase (5.8 mU mL^-1^) in a microplate well. The microplate was incubated at room temperature for 20 min and the absorbance was determined at 450 nm. The GST activity was quantified according to the method of Habig et al. ^5^. Briefly, 150 µL of sample solution was mixed with 20 µL of 10 mM GSH (in 100 mM phosphate buffer, pH = 6.5), followed by adding 20 µL of 20 mM 1-chloro-2,4-dinitrobenzene (CDNB) (in ethanol, freshly made). The blanks were prepared by mixing 170 µL of homogenization buffer with 20 µL of 20 mM CDNB. GST activity was measured immediately at 340 nm at each minute, during the first 5 min. The extinction coefficient for CDNB at 340 nm is 9.6 x 103 M^-1^ cm^-1^.

For the determination of GSH levels, the tissues were thawed on ice and homogenized in 5% cold sulfosalicylic acid solution (SSA). The homogenates were centrifuged (14,000 rpm) at 4°C for 10 min. The levels of GSH in the tissues were determined according to the method by Shaik and Mehvar ^6^. Briefly, the sample solution was diluted by 500-fold using a 100 mM phosphate buffer (pH 7.4, with 1 mM EDTA). Then 50 µL of samples was mixed with 100 µL of recycling reagent (0.3 mM NADPH, 0.225 mM DTNB, and glutathione reductase (1.6 U mL^-1^) in a 100 mM phosphate buffer (pH 7.4, with 1 mM EDTA). The absorbance was immediately recorded at 405 nm for 4 min.

The levels of malondialdehyde (MDA) in the samples were quantified according to the method by Scown et al. ^7^ with minor modifications. The tissues were thawed on ice and homogenized in of 100 mM HEPES buffer. The homogenates were centrifuged (1400 × g) at 4°C for 10 min. Then 100 µL of samples or standards (MDA dissolved in 2% ethanol (0.625 – 100 µM)) was mixed with 500 µL of 0.4% thiobarbituric acid (TBA) in 10% acetic acid, and was adjusted to pH 5.0 by NaOH. The tubes were incubated at 100°C for 1 h and then cooled on ice. Then 600 µL of butanol was added and the mixture was vortexed and centrifuged at 3100 × g for 10 min. Finally, 150 µL of the supernatant was transferred to a well in the microplate. The absorbance was measured at 532 nm. The level for MDA was expressed as nmol MDA mg protein^-1^.

For the determination of AChE activity, the tissues were thawed on ice and homogenized in 0.5 M Tris-HCl (pH 8.0) using a Pestles in G-Tubes® 1.5-mL homogenizer. The homogenates were centrifuged (1,000 × g) at 4°C for 10 min. The AChE activity was determined by a method modified from Assis et al. ^8^. Briefly, 20 µl of samples or the blanks (0.5 M Tris-HCl buffer, pH 8.0) were added to 200 µl of 0.25 mM DTNB (5,5’ – dithiobis (2-nitrobenzoic) acid) dissolved in 0.5 M Tris-HCl buffer (pH 7.4), and the reaction was initiated by adding 30 µl of 62 mM Acetylthiocholine iodide in the reaction mixture. The absorbance was recorded at 405 nm at each minute during 5 min.

**References**

1 Xie, L. & Buchwalter, D. B. Cadmium exposure route affects antioxidant responses in the mayfly *Centroptilum triangulifer*. *Aquat. Toxicol.* **105**, 199-205 (2011).

2 Chen, H. *et al.* Accumulation and effects of Cr(VI) in Japanese medaka (*Oryzias latipes*) during chronic dissolved and dietary exposures. *Aquat. Toxicol.* **176**, 208-216 (2016).

3 Li, L., Chen, H., Bi, R. & Xie, L. Bioaccumulation, subcellular distribution, and acute effects of chromium in Japanese medaka (*Oryzias latipes*). *Environ. Toxicol. Chem.* **34**, 2611-2617 (2015).

4 Sun, Y., Oberley, L. W. & Li, Y. A simple method for clinical assay of superoxide dismutase. *Clin. Chem.* **34**, 497-500 (1988).

5 Habig, W. H., Pabst, M. J. & Jakoby, W. B. Glutathione S-transferases. The first enzymatic step in mercapturic acid formation. *J. Biol. Chem.* **249**, 7130-7139 (1974).

6 Shaik, I. H. & Mehvar, R. Rapid determination of reduced and oxidized glutathione levels using a new thiol-masking reagent and the enzymatic recycling method: application to the rat liver and bile samples. *Anal. Bioanal. Chem.* **385**, 105-113 (2006).

7 Scown, T. M. *et al.* Effects of aqueous exposure to silver nanoparticles of different sizes in rainbow trout. *Toxicol. Sci.* **115**, 521-534 (2010).

8 Assis, C. R. *et al.* Characterization of acetylcholinesterase from the brain of the Amazonian tambaqui (*Colossoma macropomum*) and in vitro effect of organophosphorus and carbamate pesticides. *Environ. Toxicol. Chem.* **29**, 2243-2248 (2010).
